# Extensive Intracardiac and Deep Venous Thromboses in a Young Woman with Heparin-Induced Thrombocytopenia and May-Thurner Syndrome

**DOI:** 10.1155/2017/8530476

**Published:** 2017-03-30

**Authors:** Yekaterina Kim, Daniel C. Choi, Ali N. Zaidi

**Affiliations:** ^1^Montefiore Medical Center, Albert Einstein College of Medicine, Bronx, NY, USA; ^2^Montefiore Heart & Vascular Care Center, Albert Einstein College of Medicine, Bronx, NY, USA

## Abstract

A 38-year-old woman with a history of recurrent deep venous thromboses (DVTs) on chronic anticoagulation presented with acute left leg swelling. The patient was diagnosed with an acute left lower extremity (LLE) DVT in the setting of May-Thurner syndrome for which treatment with unfractionated heparin was started. Her hospital course was complicated by a new diagnosis of heparin-induced thrombocytopenia (HIT), with an incidental discovery of a large tricuspid valve mobile mass on a transthoracic echocardiogram (TTE). Subsequent imaging confirmed multiple right atrial thrombi along with LLE venous stent thrombosis and a new right LE acute DVT. Anticoagulation with argatroban for HIT thrombosis was started. She underwent a right atrial percutaneous thrombectomy and bilateral lower extremity thrombectomy with directed angioplasty and stent placement. This presentation is a rare manifestation of HIT with extensive intracardiac and deep venous thrombi, with successful staged interventions.

## 1. Introduction

HIT is an adverse immune reaction to heparin characterized by systemic platelet consumption and paradoxical thrombotic complications. HIT is mediated by immunoglobulins directed against platelet factor 4 (PF4) bound to heparin, which also activate platelets leading to thrombosis [1]. The incidence of HIT in hospitalized patients is estimated at 0.2% [2]. Thrombosis secondary to HIT can rarely precipitate severe complications, from intracranial thrombi to skin necrosis and limb artery thrombosis [3]. Intracardiac thrombosis is an onerous manifestation of HIT that has received increasing attention in the current case literature. We report a case of multiple large atrial thrombi associated with HIT in a patient with May-Thurner syndrome and acute bilateral deep venous thromboses (DVTs).

## 2. Case Report

A 38-year-old woman with a history of recurrent LLE DVTs in the setting of pregnancy and travel presented with new left leg swelling. She had a negative hypercoagulable workup and was previously treated with rivaroxaban. She had previous exposure to unfractionated heparin with no complications. Heparin infusion was started for her new LLE DVT. CT imaging revealed effacement of the left common iliac vein by the right common iliac artery suggestive of May-Thurner syndrome. Subsequent venogram revealed complete occlusion of the left external iliac vein and left common femoral vein for which thrombolysis with tissue plasminogen activator (tPA) was administered, followed by angiography with stent placement.

**Figure 1:**
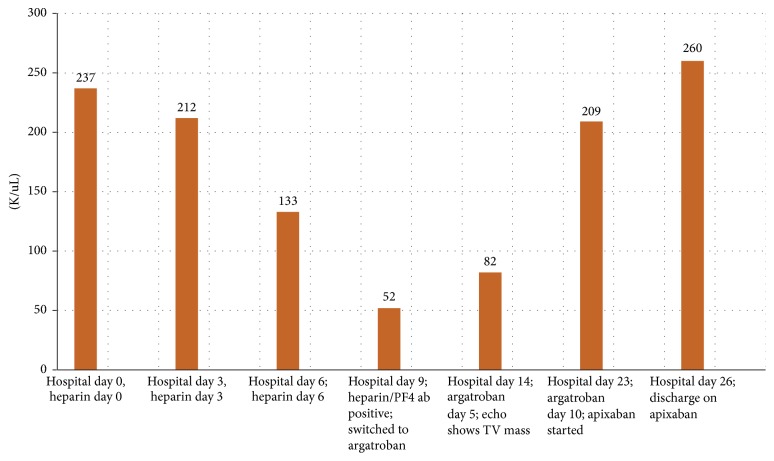
Timeline of platelet counts.

**Figure 2:**
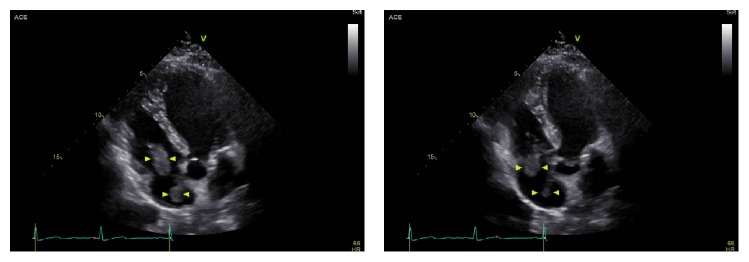
TTE showing the mobile right atrial mass that was traversing the tricuspid valve.

**Figure 3:**
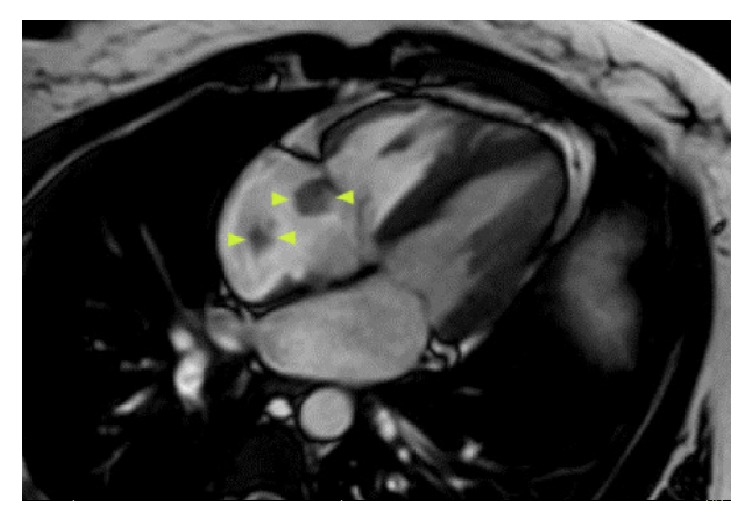
Cardiac MRI image showing multiple right atrial thrombi.

The patient's hospital course was complicated by a 44% decline in platelet count on day 6 of heparin therapy (baseline: 237K/*μ*L to 133K/*μ*L). Platelets continued to decline on heparin to a nadir of 52K/*μ*L on hospital day 9 (Figure 1). At that time, her anticoagulation was switched to argatroban and testing revealed positive heparin/PF4 antibody by IgG ELISA (OD 2.502, Quest Diagnostics) and positive serotonin release assay (100% release at UFH 0.1 IU/mL and 0.5 IU/mL, Quest Diagnostics). The patient was asymptomatic until a presyncopal episode on hospital day 14. TTE revealed a large mobile mass adherent to the tricuspid valve (Figure 2). A cardiac MRI revealed multiple right atrial thrombi (Figure 3). She was also noted to have LLE stent occlusion and an acute right lower extremity DVT. The patient was transferred to the cardiac intensive care unit (CICU) where she remained hemodynamically stable and asymptomatic. The patient subsequently underwent right atrial thrombectomy via AngioVac, revealing organizing thrombi on surgical pathology, with resolution of her clot burden on follow-up TTE. Her bilateral DVTs were treated with bilateral ultrasound-enhanced tPA thrombolysis followed by mechanical thrombectomy, angioplasty, and stent placement. After procedure, venogram showed significantly improved venous flow through both lower extremities. She was started on apixaban and discharged home without complications.

## 3. Discussion

Heparin-induced thrombocytopenia is a rare but serious complication of heparin therapy, with incidence of HIT being 3% to 5% with unfractionated heparin and less than 1% with low-molecular-weight heparins [4]. While bleeding is justifiably the complication of foremost concern to clinicians in the setting of heparin use, thrombotic complications can be equally fatal and must also be considered. The patient in our case was found to have extensive HIT thrombosis with multiple large intracardiac thrombi and acute bilateral lower extremity DVTs. Given her clot burden, she was at high risk of pulmonary embolism and cardiopulmonary compromise, therefore requiring prompt interventions to reduce her morbidity and mortality risk.

HIT encompasses multiple clinical syndromes and can include nonimmune etiologies of thrombocytopenia as well. However, the activation of platelets by immunoglobulins (usually IgG) against PF4/heparin complexes is the pathophysiologic mechanism of greatest clinical concern [5]. Platelet-activating PF4/heparin IgG complexes typically form within 5–15 days of exposure to heparin and are associated with a 20–50% risk of thromboembolic events and a mortality rate of 20% [5]. Previous reports of intracardiac thrombi in the setting of HIT have described critically ill patients, often with cardiogenic shock or arrhythmias, which themselves can predispose to the formation of intracardiac thrombi [6, 7]. Remarkably in this case, the patient remained hemodynamically stable and asymptomatic, with no sign of cardiopulmonary compromise. Her case serves to reinforce the importance of a low threshold to discontinue heparin for alternative therapeutic anticoagulation in the setting of thrombocytopenia regardless of the patient's overt clinical state.

The use of novel oral anticoagulants (NOAC) in the management of HIT is an emerging subject of study. Clinical experience, though as yet limited, has shown good platelet count responses and tolerability in patients with HIT transitioned to NOAC, both directly after heparin discontinuation and after argatroban therapy [8, 9]. Our patient received a total of 10 days of argatroban therapy while hospitalized; her platelet count had recovered to 209K/*μ*L when apixaban was started for lifelong anticoagulation given her risk for recurrent DVT. She received a loading dose of 10 mg twice daily for 7 days, transitioned as an outpatient to a maintenance dose of 5 mg twice daily. She remained without evidence of recurrent thrombosis at her 2-week and 3-month follow-ups.

This patient was incidentally found to have an anatomic predisposition to venous thrombi when CT revealed evidence of left common iliac vein compression against her fifth vertebral body by her right common iliac artery. This finding is suggestive of May-Thurner syndrome, a pathologic condition characterized by LLE venous insufficiency secondary to left iliac vein compression. As the compression is usually physiologic, May-Thurner syndrome is rarely considered during the workup of recurrent DVTs; indeed the syndrome presents as DVT in only 2-3% of cases [10]. However, retrospective analysis of CT scans suggests prevalence of up to 24% [11], so May-Thurner syndrome remains an important consideration in young patients with recurrent DVTs without other localizing predispositions to thrombosis.

Studies suggest that endovascular correction of pathologic insufficiency via venous stenting may improve symptoms [12, 13]. Preexisting thrombi and thrombophilia can affect the technical success and outcomes of endovascular venous stenting. Our case illustrates the failure of an initial venous stenting intervention in the setting of active HIT, followed by the success of a staged endovascular intervention, where preexisting HIT-induced thrombi were treated by atrial thrombectomy before ultrasound-enhanced tPA thrombolysis, angioplasty, and stenting were completed.

HIT is an important consideration in patients hospitalized for thrombotic events with the delayed onset of thrombocytopenia while on heparin therapy. Despite the onerous potential consequences of HIT in this patient with extensive thrombosis in the setting of an anatomical predisposition to DVT, her relatively young age and excellent baseline functional capacity facilitated successful staged endovascular management of both her intracardiac thrombi and her acute DVTs.

## Abbreviations

CICU: Cardiac intensive care unit
CT: Computed tomography
DVT: Deep venous thrombosis
HIT: Heparin-induced thrombocytopenia
Ig: Immunoglobulin
LLE: Left lower extremity
NOAC: Novel oral anticoagulants
PF4: Platelet factor 4
SRA: Serotonin release assay
tPA: Tissue plasminogen activator
TTE: Transthoracic echocardiogram
UFH: Unfractionated heparin.
